# The unique mean seasonal cycle in the Indian Ocean anchors its various air-sea coupled modes across the basin

**DOI:** 10.1038/s41598-021-84936-w

**Published:** 2021-03-11

**Authors:** Xinqiang Xu, Lei Wang, Weidong Yu

**Affiliations:** 1 First Institute of Oceanography, and Key Laboratory of Marine Science and Numerical Modeling, Ministry of Natural Resources, Qingdao, 266061 China; 2 Laboratory for Regional Oceanography and Numerical Modeling, Pilot National Laboratory for Marine Science and Technology, Qingdao, 266071 China; 3 Shandong Key Laboratory of Marine Science and Numerical Modeling, Qingdao, 266061 China; 4 School of Atmospheric Sciences, Sun Yat-Sen University, Zhuhai, 519082 China; 5 Key Laboratory of Tropical Atmosphere-Ocean System (Sun Yat-Sen University), Ministry of Education, Zhuhai, 519082 China; 6 Guangdong Province Key Laboratory for Climate Change and Natural Disaster Studies, Sun Yat-Sen University, Zhuhai, 519082 China; 7 Southern Marine Science and Engineering Guangdong Laboratory (Zhuhai), Zhuhai, 519082 China

**Keywords:** Physical oceanography, Physical oceanography

## Abstract

The interannual variability of the sea surface temperature (SST) in the Indian Ocean is complex and characterized by various air-sea coupled modes, which occur around El Niño/La Niña's peak phase (i.e. December–January–February, DJF). Indian Ocean Dipole Mode (IODM) develops over the tropical Indian Ocean and peaks in September–October–November (SON), while Ningaloo Niño, Subtropical Indian Ocean Dipole (SIOD) and Indian Ocean Basin Mode (IOBM) occur respectively over northwest off Australia, subtropical and tropical Indian Ocean, during boreal winter to spring. The apparent contrast between their divergent regionality and convergent seasonality around DJF triggers the present study to examine the interaction between the local mean monsoonal cycle and the anomalous forcing from El Niño/La Niña. The diagnosis confirms that the Indian Ocean’s unique complexity, including the monsoonal circulation over the tropics and the trade wind over the subtropical southern Indian Ocean, plays the fundamental role in anchoring the various regional air-sea coupled modes across the basin. The SST anomalies can be readily explained by the wind-evaporation-SST (WES) mechanism, which works together with other more regional-dependent dynamic and thermodynamic mechanisms. This implies that El Niño/La Niña brings much predictability for the Indian Ocean variations.

## Introduction

Tropical Ocean plays a critical role in regulating the weather and climate from local to global scales via the rigorous air-sea interactions over the warm water. The tropical Pacific hosts the most significant air-sea coupled mode El Niño and Southern Oscillation (ENSO)^1–3^, which occurs irregularly every 2–7 years with the far-reaching impacts across the globe^4,5^ and even to stratosphere^6^. It also has a meridional air-sea coupled mode^7^ independent from ENSO. The air-sea interactions in the tropical Atlantic Ocean shares the similarity with those in the tropical Pacific Ocean, including the zonal mode Atlantic Niño^8^ and the meridional mode^7,9^. It is further identified that the coastal Niño/Niña events develop along the eastern boundary upwelling coasts in the subtropical Pacific and Atlantic Oceans, including the California Niño/Niña^10–12^ and Chile Niño/Niña^13^ in the subtropical eastern Pacific Ocean, and the Benguela Niño/Niña^14^ and Dakar Niño/Niña^15^ in the subtropical eastern Atlantic Ocean.

Contrasting to the trade wind prevailing Pacific and Atlantic Oceans, the Indian Ocean is not only small in size but also characterized by the mixed nature of monsoon regime over the tropics and trade wind regime over the subtropical Southern Indian Ocean. It is traditionally regarded as the slave ocean of the Pacific Ocean and hence received less attention until the Indian Ocean Dipole Mode (IODM)^16,17^ was proposed. Recent works have highlighted the spatially and temporally rather complex air-sea coupled modes as reviewed in^18^, including IODM over the tropical Indian Ocean with its peak season in boreal autumn, the Indian Ocean Basin Mode (IOBM) over the northern Indian Ocean occurring in boreal spring^19–21^, the subtropical Indian Ocean Dipole Mode (SIOD) over the southern Indian Ocean in boreal spring^22^ and the most recently discovered mode called Ningaloo Niño off the northwestern coast of Australia during the boreal winter^23^.

The previous studies have revealed the governing mechanisms of the above Indian Ocean modes. IOBM is mainly attributed to the remote El Niño forcing via the atmospheric bridge^19–21^. There is a growing consensus that the downwelling Rossby wave caused by anticyclonic wind anomalies in southeastern tropical Indian Ocean propagates westwards and results in the sea surface temperature anomaly (SSTA) in the southwestern Indian Ocean^24,25^, which further increases the tropical Indian Ocean SST^26^. IODM develops through the Bjerknes air-sea feedback^16,17,27,28^, which could be triggered by El Niño or other sources. SIOD is caused by the strengthening and southward movement of Mascarene high^22,29^, and also affected by air–sea coupling mainly through the mixed layer thermodynamics^29,30^. Ningaloo Niño maintains itself through the local air-sea interactions while it is often triggered by La Niña or other factors^31–33^. Following La Niña, the Indonesia Throughflow (ITF) and equatorial waveguide processes contribute significantly to Ningaloo Niño development^31^.

Two intrinsic questions not well answered yet are: (1) why does the Indian Ocean basin even with the mixed regime of monsoon and trade wind, hosts the similar air-sea coupled modes with Pacific and Atlantic Oceans? (2) why do the Indian Ocean air-sea coupled modes exhibit the broad spatial regionality but converge in their temporal seasonality around DJF? These essential questions are addressed here by taking account of complex background atmospheric circulation patterns in the Indian Ocean, including the monsoonal circulation north of 10°S and the trade wind regime south of 10°S. The tropical Indian Ocean is featured by the most prominent monsoon on earth, exhibiting the wind from the tropical ocean to the Asian continent during the boreal summer and the reversed wind in the boreal winter^18^. The southern Indian Ocean is dominated by the southeasterly trade wind, sharing the similarity with other basins. The mixed nature of monsoon and trade wind regimes in the Indian Ocean basin provides the breeding ground for the high variability to be diagnosed below.

Using the sea surface wind data from NCEP-DOE Reanalysis2, sea surface temperature (SST) data from NOAA Optimum Interpolation SST (OISST), and wind speed and latent heat flux from Objectively Analyzed air-sea Fluxes (OAFlux), the air–sea coupling processes are diagnosed to gain the in-depth understanding of SSTA complexity from the point of view of mean state-perturbation interaction.

## Result

**Figure 1:**
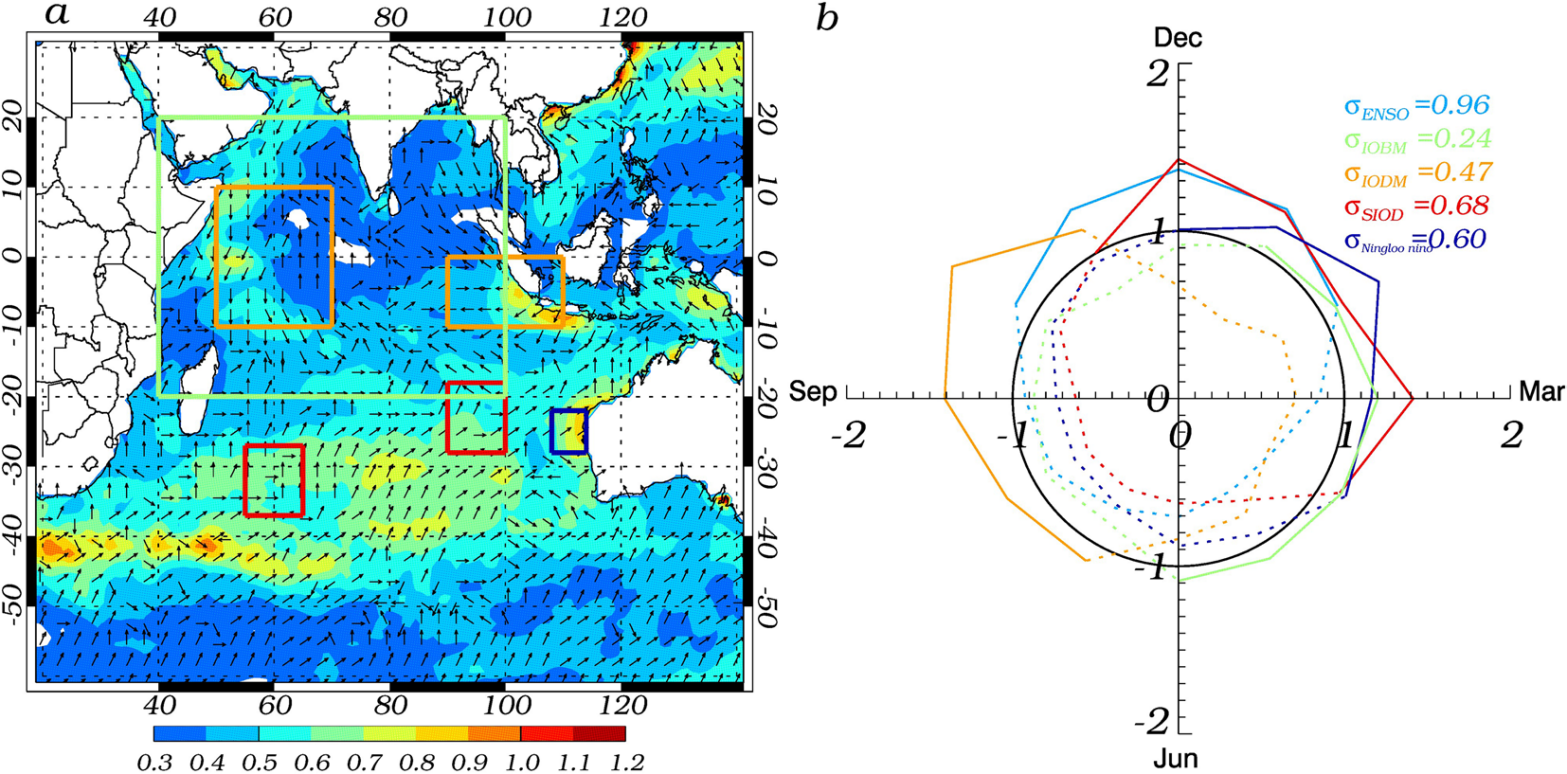
(**a**) The temporal and spatial distribution of maximum standard deviation of monthly mean SST (℃) in the Indian Ocean during 1982–2018 (the arrow indicates the month in which the maximum standard deviation occurs, increase clockwise, pointing north for December, pointing right for March). (**b**) The respective normalized monthly standard deviation of indexes, including those of El Niño and Southern Oscillation (ENSO) (light blue ), Indian Ocean Basin Mode (IOBM) (green), Indian Ocean Dipole Mode (IODM) (orange), Subtropical Indian Ocean Dipole (SIOD) (red) and Ningaloo Niño (dark blue). The solid (dashed) lines indicate the monthly standard deviation of indexes higher (lower) than one standard deviation.

It is always helpful to start from looking at the general SST variability pattern in the Indian Ocean. Figure 1a shows the spatial pattern of maximum values of the monthly SST standard deviation in shaded color and its corresponding month in unit vector, which represents December while pointing north and marches clockwise as calendar month. The regionality of intensive SST variability is clearly identified at several key regions, characterized by the rectangular boxes. These significant signals represent the strong SST interannual variations, which are potentially the consequence of the ocean–atmosphere interactions. Overall, there are four key regions with the significant SST variability, which are discussed below.

For the tropical Indian Ocean, the strong SST variability stands out over the western and eastern ends and this east–west dipole structure is understood to be attributed to IODM^16,17^. To capture the temporal phase-locking character, the monthly normalized IODM index, which is scaled by its overall standard deviation, is given in Fig. 1b. It is clear that IODM exhibits the quick development from June, peaks during September–October–November (SON) and decays dramatically in boreal winter. It is noted that the eastern and western poles have slightly different temporal peaking time. Figure 1 depicts well the seasonality of IODM evolution.

The second prominent SST variability (represented by the dark blue box in Fig. 1a) appears off the northwestern coast of Australia, which is understood to be attributed to Ningaloo Niño^23^. The monthly standard deviation of Ningaloo Niño index, defined as the averaged SST anomaly over the dark blue box, is shown in Fig. 1b. It is observed that Ningaloo Niño is phase-locked to January and February, which is consistent with the vector information in the dark blue box from Fig. 1a.

The third outstanding SST variability region goes beyond the tropical Indian Ocean and is located in the southern Indian Ocean (Fig. 1a). Previous research has identified that this vast southern Indian Ocean SST variability is associated with SIOD^22^, whose index can be defined as the difference between SST anomalies averaged over two red boxes in Fig. 1a. Its seasonality exhibits a slightly bimodal feature peaking over December and March respectively as shown in Fig. 1b.

Lastly, it is understood that the tropical Indian Ocean also hosts the so-called IOBM^19–21^, whose representative index can be defined as the box-averaged SST anomalies over the green box in Fig. 1a. Even though the consistent SST variations within this green box is not significant from Fig. 1a, this IOBM is included here from the sake of the completeness. Its peak is known to be in March from Fig. 1b.

**Figure 2:**
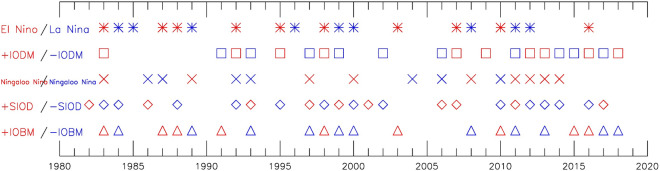
The positive (red) and negative (blue) phase events identified from their respective index during 1982–2018. For convenience of comparison, ENSO (IODM) events are labeled as the decaying (second) year.

It is interesting to observe the different regionality and seasonality associated with the above four modes in Fig. 1, with reference to the ENSO development represented by its normalized monthly standard deviation of Niño-3.4 index in Fig. 1b. It is already known that ENSO is the one of the most important external forcing of the Indian Ocean variability. With reference to the time series of the Niño-3.4, IODM, IOBM, Ningaloo Niño and SIOD indexes are plotted (shown in Fig. S1). Accordingly, the individual events are identified and summarized in Fig. 2 and their statistical characteristics are summarized in Table 1, where the bold/italic means the positive/negative phase. IODM likely occurs in ENSO's developing phase, while the other three modes in ENSO's decaying phase. With reference to ENSO cycle, the four Indian Ocean modes exhibit strong asymmetry between their positive and negative phases. Some strong linkages are identified here for further analysis, while the asymmetry itself is beyond of the scope of the present work. The positive IODM, positive Ningaloo Niño and negative SIOD are emphasized here, considering their significant linkage with the tropical Pacific variability. During the El Niño developing phase, more than half (6 from 10) of the positive IODMs occur. In the El Niño decaying phase, more than half (7 from 13) of the negative SIODs occur. In the La Niña decaying phase, more than half (5 from 9) of the Ningaloo Niño occur. IOBM does not exhibit asymmetry with more than half (7 from 9)/(6 from 11) of the positive/negative IOBMs occurring together with El Niño/La Niña process.

**Table 1:** The classification of Indian Ocean modes with reference to ENSO phases.

	El Niño	La Niña	None
IODM (**10**/*10*)	**6**/*0*	**1**/*2*	**3**/*8*
Ningaloo Niño (**9**/*7*)	**1**/*3*	**5**/*0*	**3**/*4*
SIOD (**10**/*13*)	**1**/*7*	**2**/*3*	**7**/*3*
IOBM (**9**/*11*)	**7**/*0*	**0**/*6*	**2**/*5*

Bold/italic indicates the positive/negative phase.

Then the question that follows immediately is why are these four modes arranged so reasonably in space and time, in concert of ENSO life cycle? The answer potentially lies in how the unique local seasonal cycle in the Indian Ocean interacts with the external forcing from ENSO. The intriguing fact is that even the external forcing from ENSO keeps almost the steady loading over the Indian Ocean during its developing, peaking and decaying phases, the consequence of ocean–atmosphere interaction process can be very different because the Indian Ocean has its dramatically changing monsoonal circulation. With the reversing monsoonal circulation, the actual wind (the sum of the mean wind and the anomalous wind) can be enhanced in one season but reduced in another season. This means the air-sea coupled modes can be maintained only during some specific time window and over some specific region. Hence the monsoon seasonal cycle plays the critical role in geographically anchoring the various air-sea coupled modes across the basin, which are all convergent towards ENSO peak time.

**Figure 3:**
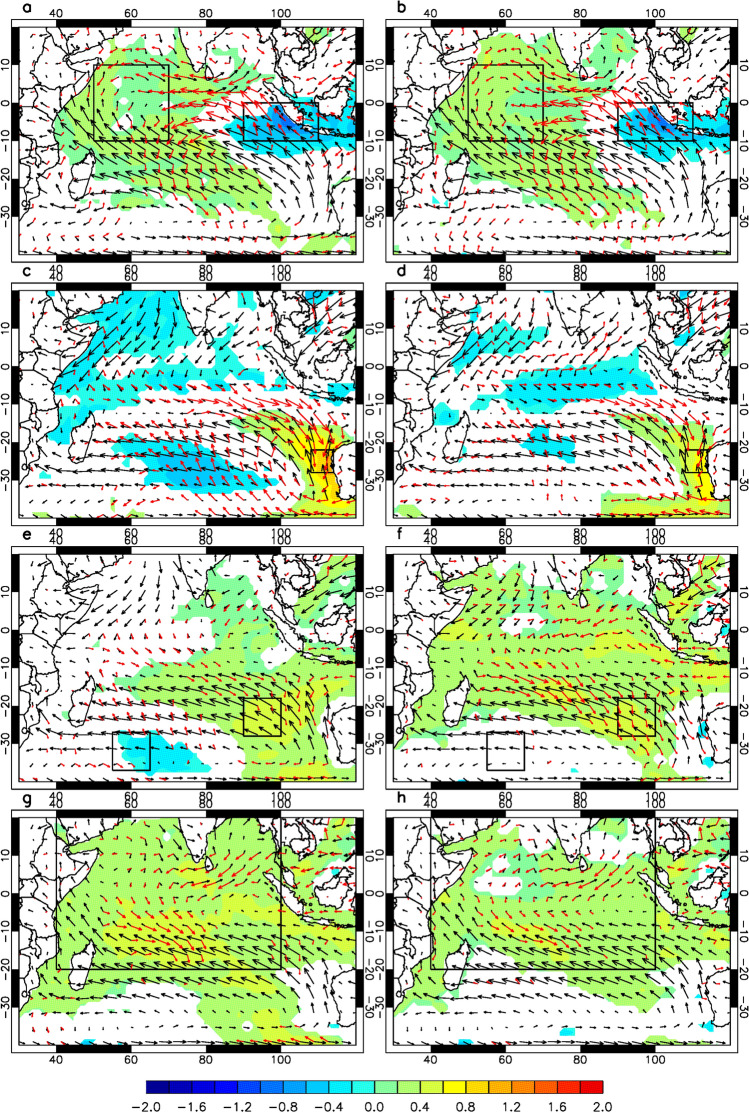
The composite maps of anomalous SST and 10 m wind field during, (**a**) Sep.–Nov. for all positive IODMs, (**b**) same as (**a**) except for El Niño, (**c**) Jan.–Mar. for all positive Ningaloo Niño, (**d**) same as (**c**) except for La Niña, (**e**) Feb.–Apr. for all negative SIODs, (**f**) same as (**e**) except for El Niño, (**g**) Mar.–May for all positive IOBM, (**h**) same as (**g**) except for El Niño. The climatological /anomalous winds are is indicated by black/red arrows. The regions with the SST anomalies exceeding 90% confidence level are shaded, and red arrows exceed 90% confidence level.

The composite analysis is adopted here to confirm if the Indian Ocean changing monsoonal seasonal cycle can set up its different air-sea couple modes in response to the remote forcing from ENSO. Following the classification in Table 1, the composite maps of anomalous SST and 10 m-height wind for the positive IODM, Ningaloo Niño, negative SIOD and positive IOBM are shown in Fig. 3. To highlight the interaction between the mean and the anomalous wind, the climate monsoonal wind is plotted in black arrow while the anomalous wind is in red. This helps the readers easily identify if the anomalous wind enhance/reduce the mean wind, which furthermore leads to the negative/positive SST anomaly through the latent heat release process, termed as wind-evaporation-SST (WES) mechanism^27^. This WES mechanism can work together with other more region-specific dynamic (e.g. wind-driven upwelling along Java-Sumatra coast) and thermodynamic (e.g. mixed layer entrainment) mechanisms.

**Figure 4:**
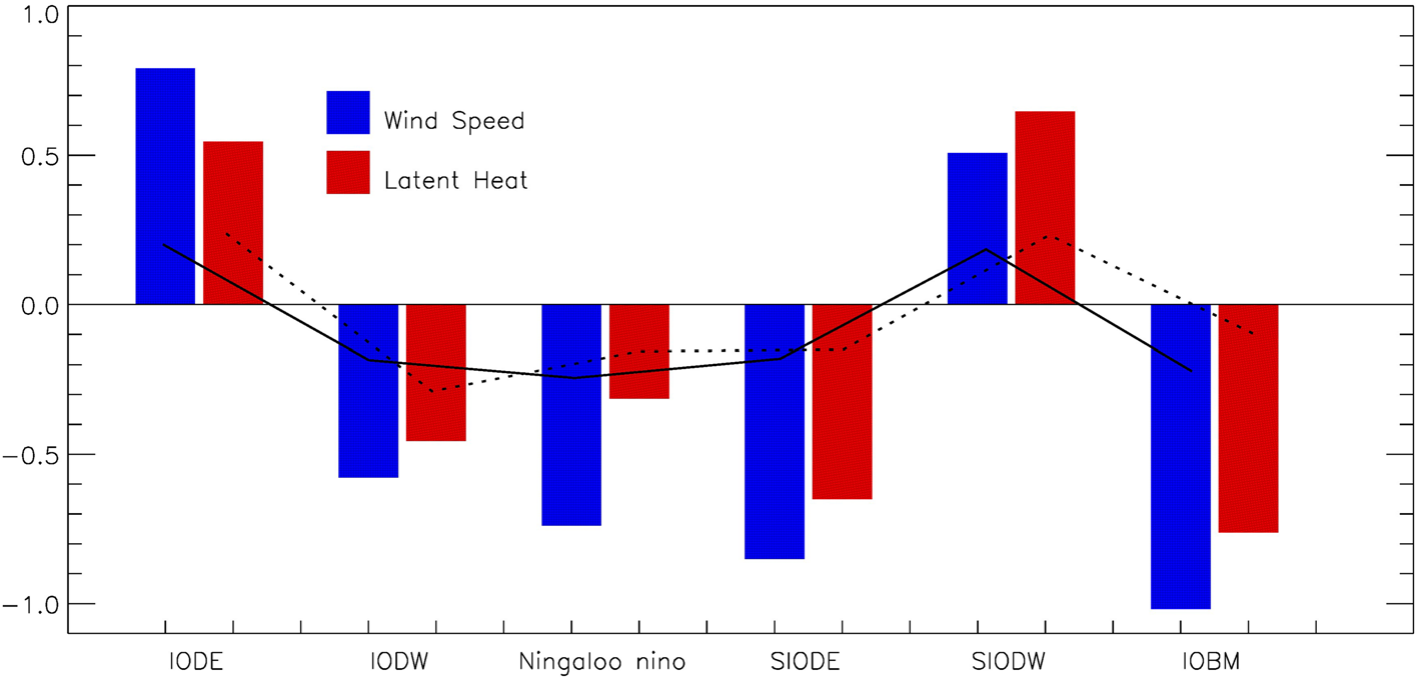
The composites of normalized (by the standard deviation of the total time series) anomalous daily 10 m-height wind speed and sea surface latent heat flux averaged over the respective boxes of the air-sea modes during the SST's fastest increasing/decreasing period (31 days) in the development and mature phases. The 90% confidence levels are plotted by solid (dashed) lines for wind speed (latent heat flux) and the daily data are applied a 11-day running mean.

The popularity of WES mechanism in the four Indian Ocean air-sea modes is further shown in Fig. 4, where the composites of the changes in wind speed and latent heat flux during their significant development stage are given. The time series of wind, latent heat flux and SST variations are given in Fig. S2. This indicates the influence of wind speed on the changes in latent heat flux and hence the triggering impact from ENSO forcing. Here the focus is given on the triggering process and the following up development mechanism via air–sea coupling is not further addressed.

The composite maps of SSTA, mean surface wind and its anomaly associated with positive IODM is firstly examined in Fig. 3a. It is understood from Table 1 that more than half of the positive IODM events occur along with El Niño and hence the corresponding composite map for all El Niño events is given in Fig. 3b for reference. It is clearly shown that the anomalous wind is almost in the same/opposite direction over the tropical eastern/western Indian Ocean during the boreal summer monsoon season, resulting in enhanced/decreased surface wind speed. The enhanced/decreased surface wind speed has its thermodynamic and dynamic consequence changing SST. The thermodynamic process is realized through WES. The dynamic effect works over the upwelling region such as in the eastern equatorial Indian Ocean offshore Java and Sumatra, which is thoroughly discussed for the case of IODM development^28,34^. The interaction between the mean and anomalous winds is very stable in the composite IODM and that of El Niño. In Fig. 4, as the wind speed in IODM eastern pole (IODE) increases, the latent heat flux increases, while it is opposite in IODM west pole (IODW). The enhanced alongshore wind in the southeastern tropical Indian Ocean cools SST not only through the enhanced evaporation but also through the enhanced upwelling. These thermodynamic and dynamic processes are well understood^28^.Ningaloo Niño mostly follows the decaying phase of La Niña, as shown in Fig. 3c,d. Northwest of Australia, the mean austral summer monsoon wind prevails between January and March. This monsoonal wind is reduced by the anomalous wind and hence the local warm SSTA develops through the reduction of latent heat release. The potential contribution from the relaxed upwelling in response to the reduced alongshore wind needs further exploration. Moving further south into the trade wind region, the southeasterly wind prevails all year round. SIOD composite exhibits the similar story (Fig. 3e,f), where the mean trade wind over the southeastern subtropical Indian Ocean is reduced by the anomalous wind. When SIOD's northeastern pole exhibits positive SSTA (as in Fig. 3e), there occurs the atmospheric cyclonic response to its left following Gill's model. The southeasterly wind anomaly in the southern part of this cyclonic circulation increases the mean southeasterly wind and hence leads to the negative SSTA over the southwestern pole of SIOD through WES mechanism. The IOBM does not show much asymmetry between its positive and negative phases with reference to ENSO cycle. The composites of the positive IOBM and El Niño are given in Fig. 3g,h. The opposite direction of mean wind and the anomalous wind mainly occurs over the southwestern tropical Indian Ocean, which is understood as the key region for IOBM.

The above analysis shows that the four variability modes (IODM, Ningaloo Niño, SIOD and IOBM) share the similar interaction between the local mean circulation and the anomalous wind from ENSO remote forcing, which helps trigger the initial SST anomalies through WES mechanism. In the upwelling regions, such as the equatorial eastern Indian Ocean and northwestern Australian coast, the ocean dynamics can further amplify the SST anomalies. The point here is that the interaction between the mean state (the mixed nature of the alternating monsoonal circulation over the tropics and the trade wind regime in the subtropical southern Indian Ocean) and the ENSO related remote forcing triggers the various air-sea coupled modes in the specific seasons and anchors them in the specific regions. The earlier study^35^ has identified that El Niño’s remote forcing over the Indian Ocean is characterized by the pair of anti-cyclonic wind curls in the eastern Indian Ocean during its developing phase till the next spring. This anticyclonic wind covers the tropical and subtropical southern Indian Ocean. Along with the seasonal cycle in the Indian Ocean, the interaction between the mean state and the external forcing exhibits different loading on SST. During the boreal summer, the southeasterly monsoon wind prevails in the tropical southern Indian Ocean. El Niño-induced anti-cyclone over the eastern Indian enhanced the wind off Java and Sumatra and hence helps trigger IODM (Fig. 3a,b). During El Niño peak phase, the northwesterly wind anomaly of the lower branch of the anti-cyclone is against the southeasterly trade wind in the southern Indian Ocean, where its helps to trigger the negative SIOD (Fig. 3e,f). Similarly, La Niña induces the cyclonic anomalies over the southeastern Indian Ocean. During the peak DJF season, its branch along the Australian western coast blows against the south and southeasterly trade wind off coast of west Australia and helps trigger the Ningaloo Niño (Fig. 3c,d). IOBM follows the similar process even though its intensity is relatively weak.

## Summary and discussion

**Figure 5:**
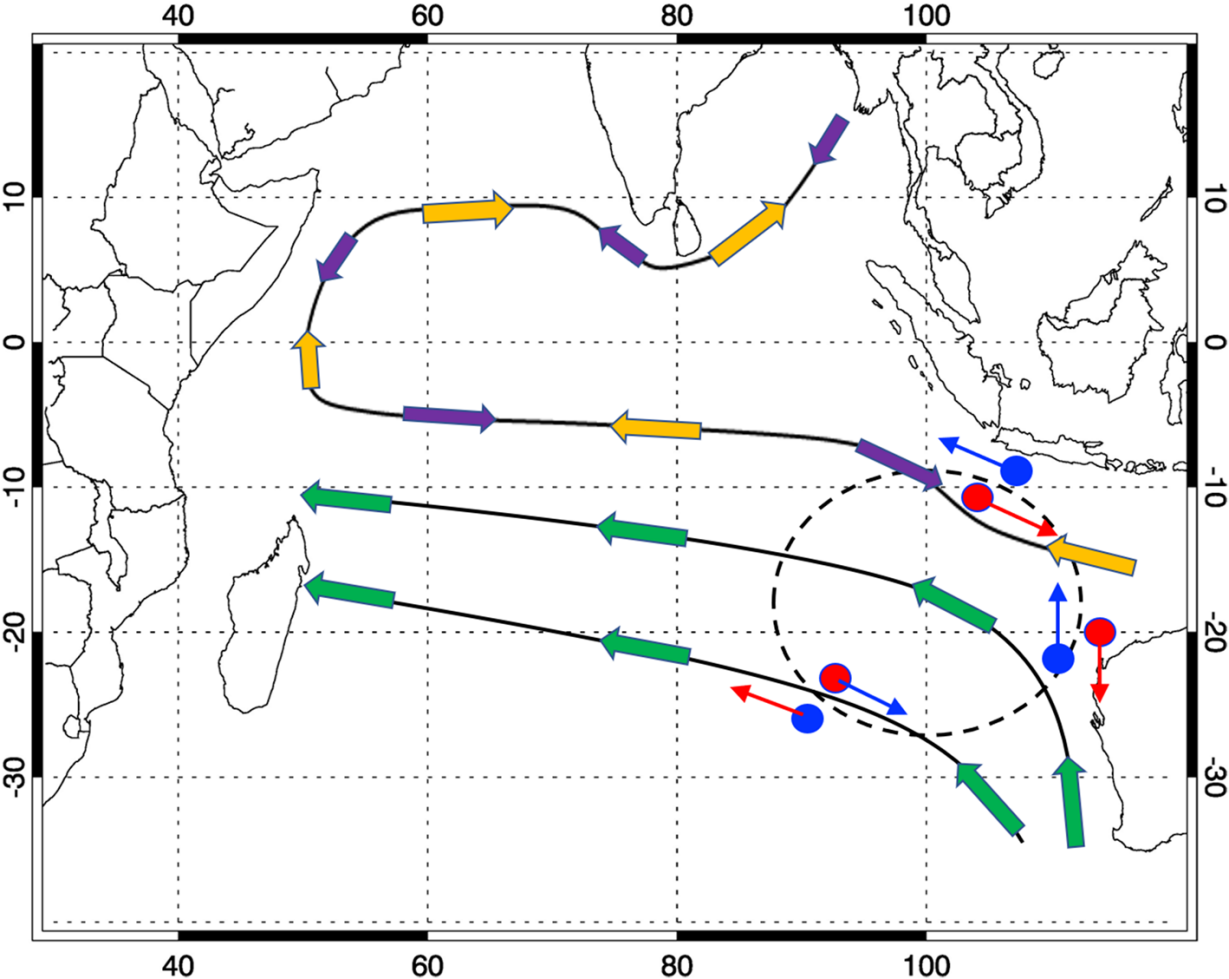
Schematic configuration of the interaction between the Indian Ocean mean state and the ENSO remote forcing. The Indian Ocean mean state is illustrated by the thick arrows, where the thick yellow (purple) arrow represents the boreal summer (winter) monsoonal circulation and the thick green arrow represents the southern Indian Ocean trade wind. El Niño/La Niña remote forcing is illustrated by the anti-cyclonic/cyclonic wind anomalies (thin blue/red arrow) over the dash-line circle region. The positive/negative SST anomaly results from the mean state-perturbation interaction is shown in red/blue dot, respectively corresponding to IODM, Ningaloo Niño/Niña and SIOD.

We here propose one uniform framework (Fig. 5) of the triggering mechanism for the various air-sea coupled modes in the Indian Ocean including IODM, IOBM, Ningaloo Niño and SIOD. The unique mean seasonal cycle in the Indian Ocean, which includes the alternating monsoon circulation over the tropics and the steady trade wind regime in the subtropics, is the fundamental cause. El Niño (La Niña), as the most important external forcing, induces the anti-cyclonic (cyclonic) wind anomaly in the southeastern Indian Ocean. These anomalies develop while ENSO evolves, loading their impacts on the Indian Ocean from the developing year (early summer) to the decay year (early summer). When the anomalous wind is in the same/opposite direction with the mean wind, it leads to the negative/positive SST anomaly through WES mechanism. Over the upwelling regions such as in the equatorial eastern Indian Ocean (northwestern Australian coast), the enhanced (weakened) alongshore wind further enhanced the SST anomaly through the ocean dynamics. The interaction between the local mean state and the external forcing helps trigger the air-sea coupled mode. As the monsoonal wind evolves with season, this interaction favors the development of the various air-sea coupled modes in the specific time window and also anchors them in the specific region.

This proposed framework helps understand the convergent seasonality around DJF and divergent regionality of the various air-sea coupled modes in the Indian Ocean. The positive IODM tends to develop during boreal summer monsoon season, when the negative SST anomaly over tropical southeastern Indian Ocean occurs as the result of the enhanced along-shore wind off Java and Sumatra. Ningaloo Niño tends to occur during the austral summer and early autumn, when the Australian monsoonal wind along the northwestern coast is reduced by La Niña's remote forcing. SIOD has the similar seasonality with Ningaloo Niño and its northeastern pole appears from the interaction between the southern branch of the El Niño induced anti-cyclone. IOBM’s SSTA mainly occurs over the southwestern tropical Indian Ocean as the result of the opposite direction of the mean wind and El Niño's remote forcing.

This proposed framework implies that the significant part of the IODM/IOBM/Ningaloo Niño/SIOD predictability originates from ENSO^36–39^. The remote forcing from ENSO helps trigger and sustain their development. Hence the better understanding on the impacts of ENSO loadings over the Indian Ocean gives insight on the complex variability in the monsoonal Indian Ocean. It should be noted that the present work emphasizes the WES mechanism in its qualitatively explaining the Indian Ocean SSTA and the mixed layer heat budget diagnosis is needed to resolve the uncertainty and further understand the thermodynamic processes quantitively, which will be explored in the next step.

## Data and method

The monthly mean SST, 10 m-height wind, wind speed and latent heat flux from 1982 to 2018 and daily data from 1985–2018 are used for the present analysis. SST is from the National Oceanic and Atmospheric Administration (NOAA) Optimum Interpolated Sea Surface Temperature (OISST) V2 dataset with 1° × 1° spatial resolution, available from https://psl.noaa.gov/data/gridded/data.noaa.oisst.v2.html. The SST data are processed using a least-square fit to remove the trend of linear change. The 10 m-height wind is from NCEP-DOE Reanalysis2, available from https://psl.noaa.gov/data/gridded/data.ncep.reanalysis2.html. The 10 m wind speed and latent flux are from WHOI OAFlux Project with 1 × 1 spatial resolution, available from http://oaflux.whoi.edu/data.html.

Here we adopt the Niño3.4 index (the averaged SSTA over the region 5°S–5°N, 170°–120°W) to represent ENSO. The IODM is described by the Dipole Mode Index (DMI), the difference of the area-averaged SST anomalies in the west pole (10°S–10°N, 50–70°E) and the east pole (10°S-Equator, 90°E–110°E). The Ningaloo Niño index (NNI) is defined by taking an area-average of SST anomalies in the box (108°E-coast, 28–22°S). The subtropical dipole index (SIOD) is obtained from the SST anomaly difference between the southwestern pole (55–65°E, 37–27°S) and northeastern pole (90–100°E, 28–18°S). The IOBM index is defined as the SSTA over the tropical Indian Ocean region (40–100°E, 20°S–20°N). All these data are applied a 3-month running mean.

## Supplementary Information

Supplementary Information
