# Supplementary material for: The unique mean seasonal cycle in the Indian Ocean anchors its various air-sea coupled modes across the basin
Source: Sci Rep. 2021 Mar 11;11:5632. doi: 10.1038/s41598-021-84936-w (PMC7970992; doi:10.1038/s41598-021-84936-w)
Supplement: Supplementary file 1 — Supplementary Information [file 41598_2021_84936_MOESM1_ESM.docx]

**The unique mean seasonal cycle in the Indian Ocean anchors its various air-sea coupled modes across the basin**

Xinqiang Xu^1,2^, Lei Wang^3,4,5,6^, Weidong Yu^*3,4,5,6^

^1^ First Institute of Oceanography, Ministry of Natural Resources, Qingdao 266061, China;

^2^ Laboratory for Regional Oceanography and Numerical Modeling, Qingdao Pilot National Laboratory for Marine Science and Technology, Qingdao 266071, China

^3^ School of Atmospheric Sciences, Sun Yat-Sen University, Zhuhai 519082, China;

^4^ Key Laboratory of Tropical Atmosphere-Ocean System (Sun Yat-Sen University), Ministry of Education, Zhuhai 519082, China

^5^ Guangdong Province Key Laboratory for Climate Change and Natural Disaster Studies, Sun Yat-Sen University, Zhuhai 519082, China

^6^ Southern Marine Science and Engineering Guangdong Laboratory (Zhuhai), Zhuhai 519082, China


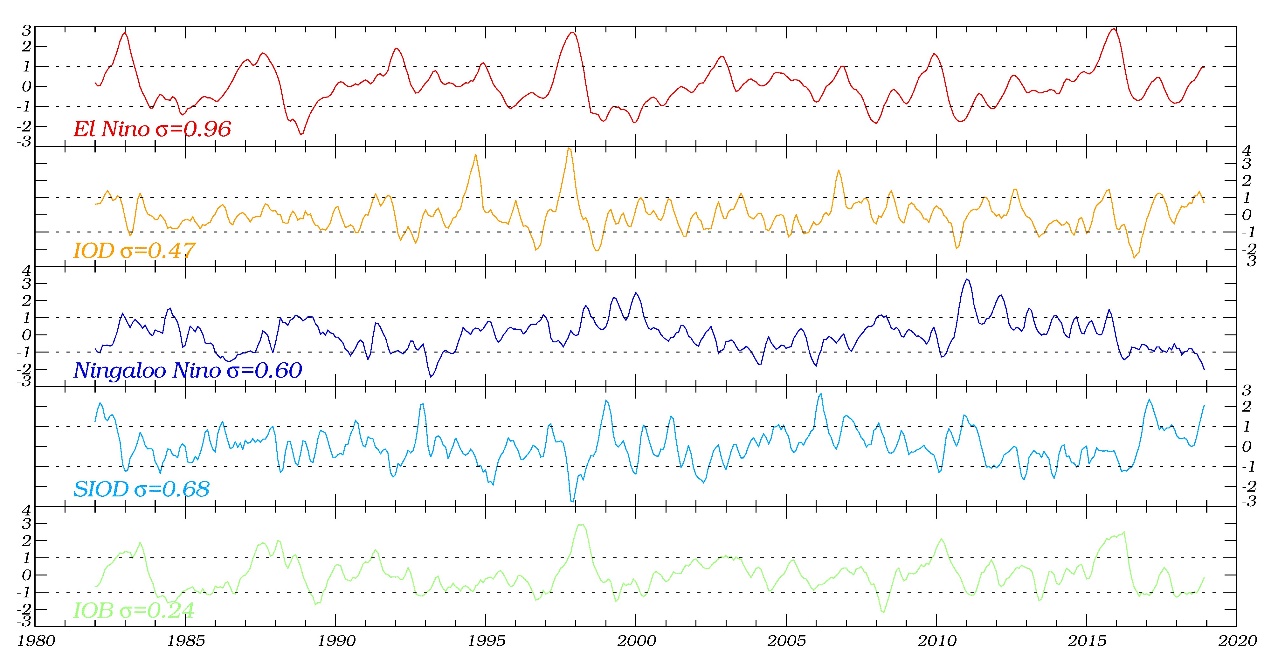


Fig.S1 The normalized time series of ENSO and IODM, Ningaloo Niño, SIOD, IOBM


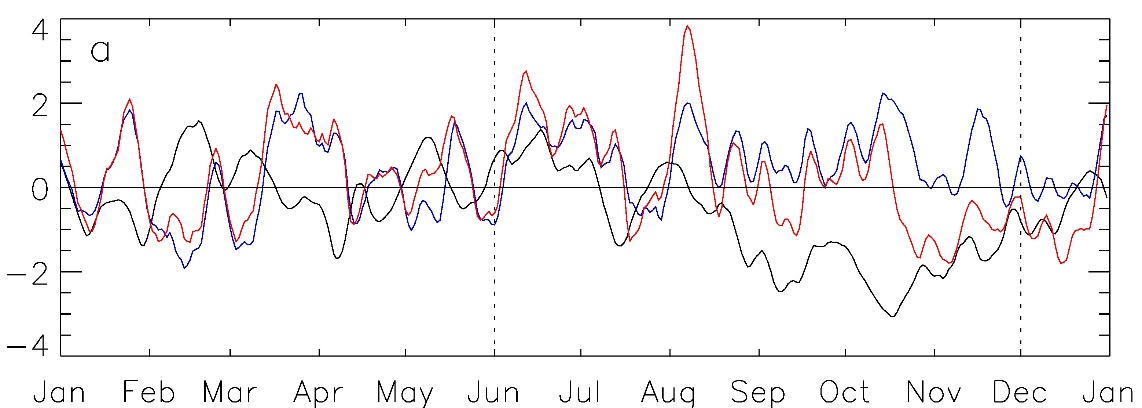


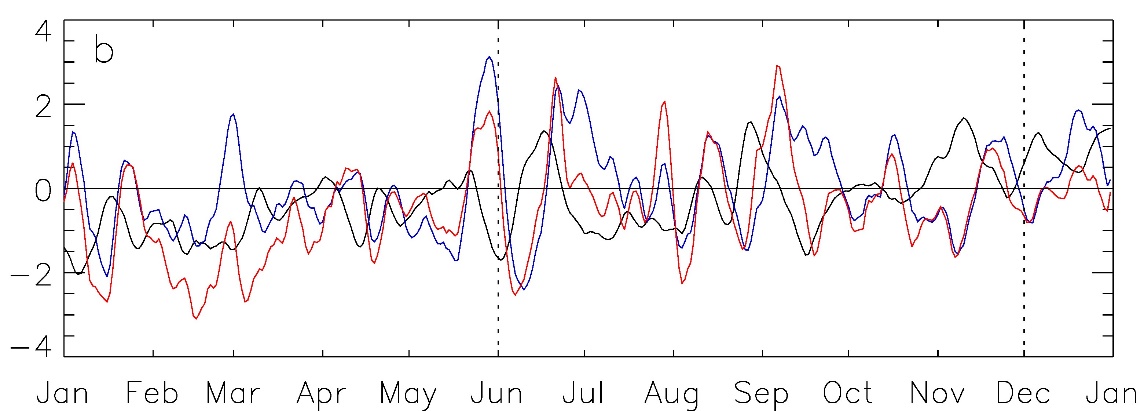


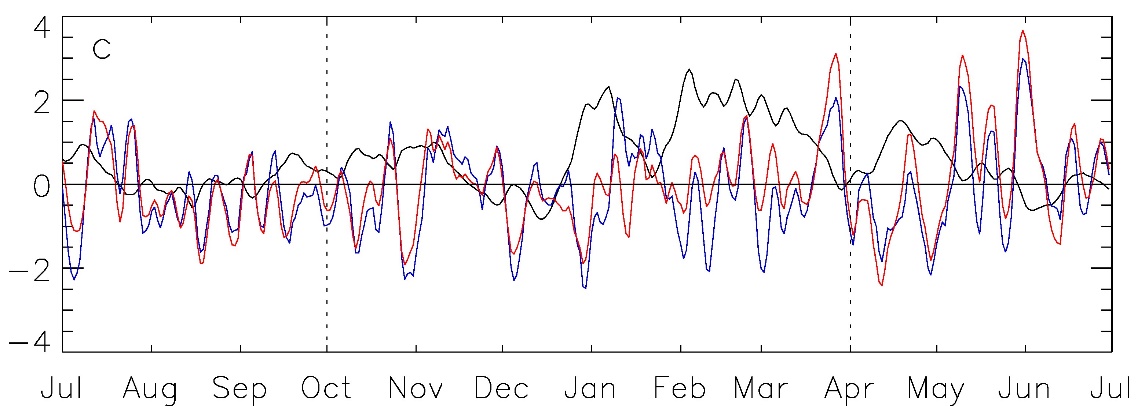


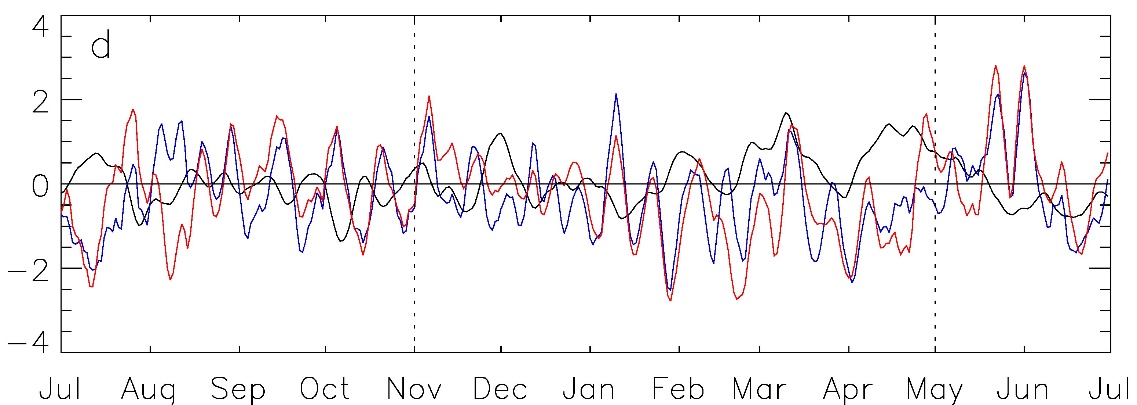


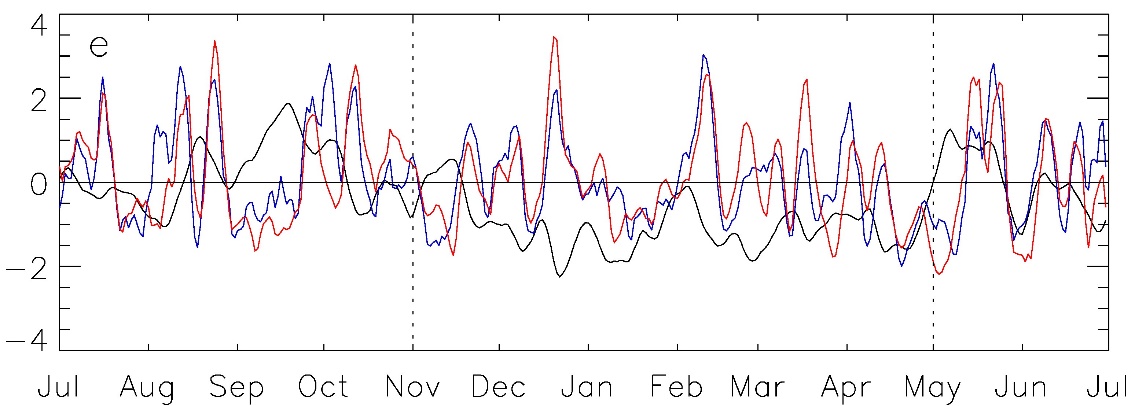


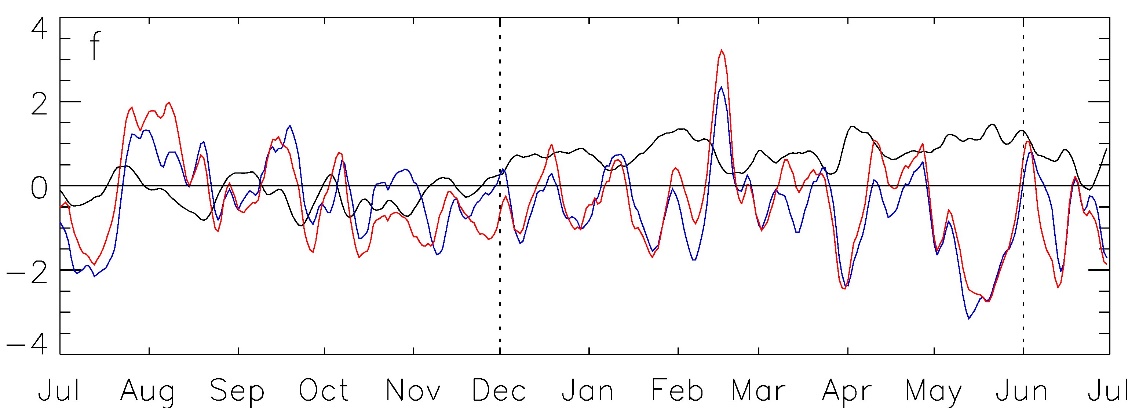


Fig.S2 The time series of normalized anomalous SST (black), wind speed (blue) and heat latent flux (red) averaged over the respective boxes, a. IODE in 2006, b. IODW in 2006, c. Ningaloo Niño in 2012/13, d. SIODE in 1991/92, e. SIODW in 1991/92, f. IOBM in 1990/91, and the daily data were applied a 5-day running mean. The development phases and mature phases are marked between two dashed lines, IODM (Jun.-Nov.), Ningaloo Niño (Oct.-Mar.), SIOD (Nov.-Apr.), IOBM (Dec.-May.), during these seasons, the SST’s fastest increasing/decreasing periods were selected for composition analysis (fig.4)
